# Sudden cardiac death diagnosed with dilated cardiomyopathy in a Kuwaiti family: a case report

**DOI:** 10.1186/1756-0500-7-914

**Published:** 2014-12-16

**Authors:** Bassam Bulbanat, Dinu Antony, Kazem Behbehani, Osama Alsmadi, Daisy Thomas, Maisa Mahmoud Kamkar

**Affiliations:** Division of Cardiology, Al-Amiri Hospital, Ministry of Health, P.O. Box 1180, Dasman, 15462 Kuwait; Genetics and Genomics Unit/Dasman Genome Center, Biomedical Research Department, Dasman Diabetes Institute, Kuwait City, Kuwait; Dasman Diabetes Institute, Kuwait City, Kuwait

**Keywords:** Cardiomyopathies, Familial dilated cardiomyopathy, Sudden arrhythmic death

## Abstract

**Background:**

Dilated cardiomyopathy is myocardial disease characterized by dilatation and impaired contraction of the left ventricle or both left and right ventricle. The majority of these cases are secondary to coronary artery disease, hypertension and valvular cardiomyopathy. Patients diagnosed with dilated cardiomyopathy are further clinically evaluated for evidence of familial history of the disease. Those families have shown to have genetic predisposition to dilated cardiomyopathy; thus, currently there is no available single genetic test that allows comprehensive testing of all causative genes. We report a Kuwaiti case of dilated cardiomyopathy that was diagnosed at young age. The patient clinical presentation pointed out to the fact that this was a familial disease. This case is the first reported in Kuwait clinically presented with familial dilated cardiomyopathy implying a genetic susceptibility factor to be further investigated within the at-risk family members.

**Case presentation:**

23-year-old Arab ethnicity Kuwaiti male with strong family history of dilated cardiomyopathy was admitted witnessed with sudden cardiac death. The patient presented with sudden arrhythmic death and survived with permanent anoxic brain injury. Transthoracic echocardiography revealed dilated cardiomyopathy with severe global left ventricular systolic dysfunction. After thorough investigation, the patient shown to have strong family history of dilated cardiomyopathy.

**Conclusion:**

Familial dilated cardiomyopathy is poorly documented in Kuwait. We present this case with future plan to study the genetic map of his family.

## Background

According to the World Health Organization (WHO), cardiovascular diseases are the number one cause of death accounting for 30 percent (17.5 million) of all deaths worldwide [1]. Cardiovascular diseases are caused by disorders of the heart and blood vessels, they includes coronary heart disease (heart attacks), cerebrovascular disease (stroke), raised blood pressure (hypertension), peripheral artery disease, rheumatic heart disease, congenital heart disease and heart failure. The WHO defined cardiomyopathies as diseases of the heart muscle. They classified them due to their etiology into, cardiomyopathies with unknown causes, and cardiomyopathies from cardiac dysfunction (sudden cardiac arrest) due to known cardiovascular disorders such as hypertension, ischemic heart disease, or valvular disease [2] (See Tables 1 and 2).

**Table 1:** **Classification criteria of primary cardiomyopathy according to the American heart association and the European society of cardiology**

Primary cardiomyopathy		
Genetic	Mixed*	Acquired
• HCM*	• DCM*	• Inflammatory (myocarditis)
• ARVC/D*	• Restrictive (nonhypertrophid and nondilated)	• Stress-provoked (*takotsubo)
• LV/NC*		• Peripartum
• Glycogen storage (PRKAG2, Danon)		• Tachycardia-induced
• Conduction defects		• Infants of insulin-dependent diabetic mothers
• Mitochondrial myopathies		
• Ion channel Disorders (LQTS, Brugada, SQTS, CVPT, Asian SUNDS)		

*HCM: Hypertrophic Cardiomyopathy, *DCM: Dilated Cardiomyopathy, *ARVC/D: Arrhythmogenic Right Ventricular Cardiomyopathy/Dysplasia, *LV/NC: Left Ventricular Noncompaction Cardiomyopathy.

**Table 2:** **Classification criteria of secondary cardiomyopathy according to the American heart association and the European society of cardiology**

Secondary cardiomyopathy	
Infiltrative	• Amyloidosis (primary familial autosomal dominant, senile, secondary forms)
	• Hurler’s disease
	• Hunter’s disease
Storage	• Hemochromatosis
	• Fabry’s disease
	• Glycogen storage disease (type II, Pompe)
Toxicity	• Drugs, heavy metals, alcohol
Endomyocardial	• Hyperesinophilic syndrome (Loeffler’s endocarditis)
Inflammatory	• Sarcoidosis
Endocrine	• Diabetes Mellitus
	• Hyperthyroidism
	• Hypothyroidism
	• Hyperparathyroidism
	• Pheochromocytoma
	• Acromegaly
Cardiofacial	• Noonan syndrome
	• Lentiginosis
Neuromuscular/neurological	• Friedreich’s ataxia
	• Duchenne-Becher muscular dystrophy
	• Emery-Dreifuss muscular dystrophy
	• Myotonic dystrophy
	• Neurofibromatosis
	• Tuberous sclerosis
Nutritional deficiencies	• Beriberi (thiamine), pellagra, scurvy, selenium, carnitine, kwashiorkor
Autoimmune/collagen	• Systemic lupus erythematosis
	• Dermatomyositis
	• Rheumatoid arthritis
	• Scleroderma
	• Polyarteritis nodosa
Consequence of cancer therapy	• Anthracyclines: doxorubicin (adriamycin), daunorubicin
	• Cyclophosphamide
	• Radiation

Cardiac deaths are classified as sudden or non-sudden, sudden cardiac death is defined as death occurring suddenly and unexpectedly in a patient who is otherwise stable prior to the event [3, 4]. Sudden cardiac death is caused by ventricular tachycardia or fibrillation and may be aborted with an implantable cardioverter defibrillator. Among young adults (18–35 years), sudden cardiac death most commonly results from a previously undiagnosed congenital or hereditary condition such as coronary artery anomalies and inherited cardiomyopathies [5]. They are classified into (i) witnessed deaths, if death occurred within 1 hour after the onset of new symptoms or as (ii) un-witnessed deaths if the patient was seen alive and stable during the previous 24-hours. On the other hand, if the sequence of events is consistent with sudden death but a specific cause of death other than arrhythmia is confirmed; the death is classified as non-sudden. A careful history and physical examination, in addition to electrocardiography and cardiac imaging, are essential to diagnose conditions associated with sudden cardiac death [6].

## Case presentation

**Figure 1:**
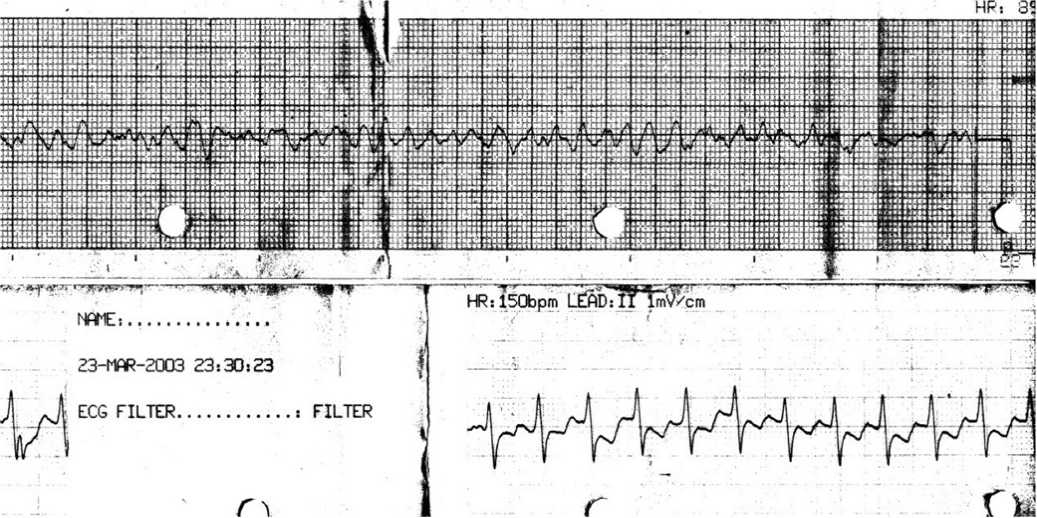
**Ventricular fibrillation of the patient using a cardiac monitor.**

**Figure 2:**
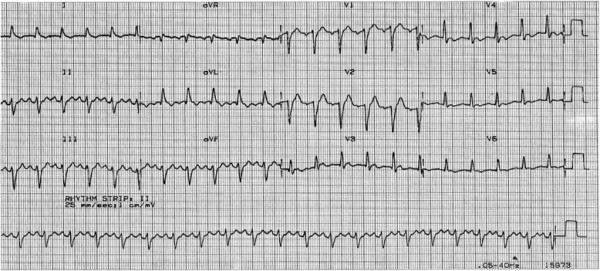
**Electrocardiography revealed sinus rhythm with incomplete left bundle branch block and non-specific ST-T wave changes.**

**Figure 3:**
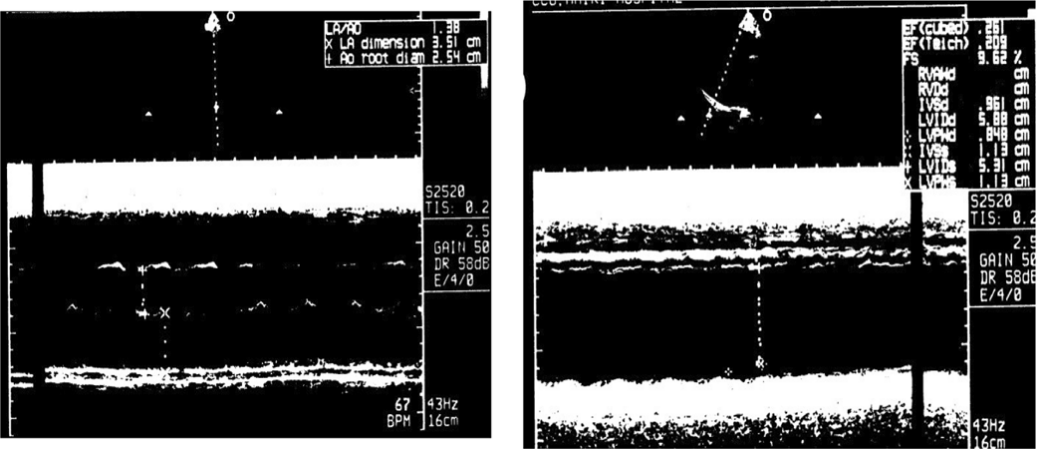
**A transthoracic echocardiography showed a mildly dilated left ventricular with severe global left ventricular systolic dysfunction with an estimated left ventricular ejection fraction of 20 to 25%.**

**Figure 4:**
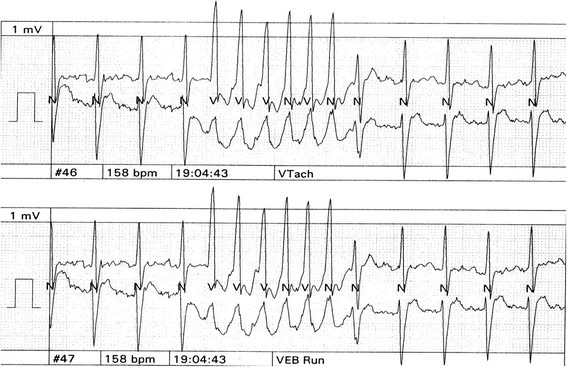
**The patient still showing non sustained ventricular tachycardia on 24 hour holter monitor.**

**Figure 5:**
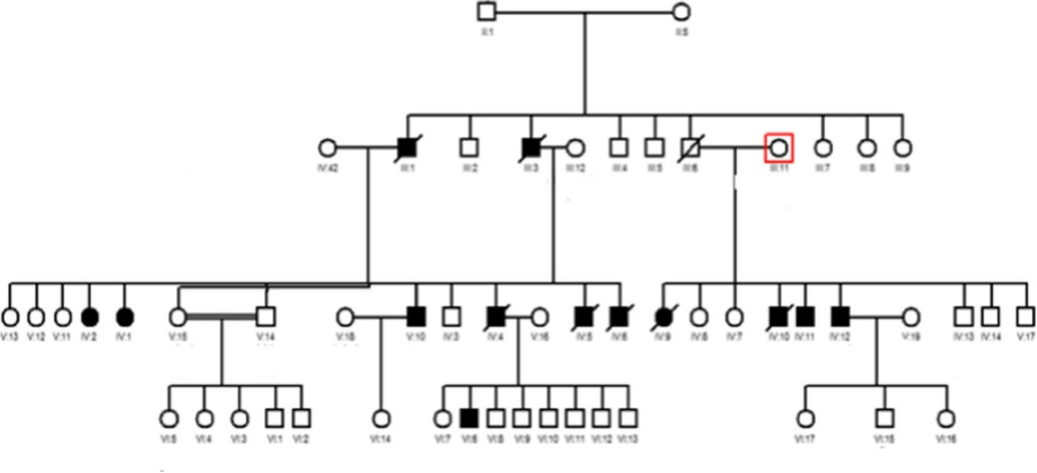
**Pedigrees of a Kuwaiti family diagnosed with familial dilated cardiomyopathy.** Squares indicate men; circles, women; crossed symbols, deceased; highlighted symbols, diagnosed with dilated cardiomyopathy and has peacemaker; clear symbol, number of unaffected/asymptomatic members.

In March 2003, a 23-year-old Arab ethnicity Kuwaiti male with a strong family history of dilated cardiomyopathy presented to a specialized neurology hospital with dizzy spells and feet parasthesia. He was given intravenous methylprednisolone for 3 days for probable multiple sclerosis and was booked electively for a magnetic resonance imaging (MRI) of the brain. On March 23^rd^ 2003, he had a witnessed sudden cardiac arrest and suddenly collapsed when he was at an automated banking teller machine. The paramedic personnel were at the scene within 5 minutes of the call, endotracheal intubation was carried out as well as an intra-tracheal atropine and adrenalin was given at the scene. A cardiac monitor was attached and revealed ventricular fibrillation (Figure 1), and thus was electrically cardioverted 3 times (200 J, 300 J and 360 J) until reverted to sinus rhythm. The resuscitation procedure including the transit time to hospital was around 30 minutes. On arrival to intensive care unit (ICU), he had a heart rate of 150 beats per minute and a blood pressure of 110/70 mm/Hg. Electrocardiography (ECG) revealed sinus rhythm with incomplete Left bundle branch block (LBBB) and non-specific ST-T wave changes (Figure 2). A transthoracic echocardiography showed a mildly dilated left ventricular (LV) with severe global LV systolic dysfunction with an estimated Left Ventricular Ejection Fraction (LVEF) of 20 to 25% (Figure 3). An echocardiogram was done 6 months prior to the hospital admission and LV systolic function reported to be normal. During his ICU stay, he had recurrent convulsions, which were controlled with epanutin and clonazepam. Computed tomography scan (CTS) of the head showed a small infarct (probably embolic) in the right centrum semiovale adjacent to the body of the right lateral ventricle. An electroencephalograph (EEG) was performed and this revealed diffuse cortical dysfunction with no epileptic discharge. He had a battery of laboratory investigations including liver function test, renal function test, complete blood count and thyroid function test and these were all within normal limits. Later on, he had a transient rise in liver enzymes and this was found to be secondary to hepatic hypo-perfusion (hepatitis viral screening, auto-immune screening and serum copper tests were all normal). Eventually a tracheostomy was performed and a nasogastric tube fed him. His neurological condition showed a slow but steady improvement over a course of 3 months and regained consciousness, with spontaneous breathing and thus the tracheostomy was closed. However, he was left mentally handicapped with sphincteric incontinence and was referred to the rehabilitation hospital for aggressive physiotherapy. During his follow up, he was treated medically with anti-failure and antiepileptic drugs (digoxin, revotril, epanutin, zestril, lasix, zantac and amiodarone). However, he continued to show non-sustained ventricular tachycardia on a 24-hour holter monitor (Figure 4). Eventually, he had an automated implantable cardioverter-defibrillator (AICD) inserted in 2004 and his functional status significantly improved and he is still alive. He developed amiodarone induced thyroiditis with hyperthyroidism, Thyroid test, FT4 = 15.29 (Normal = 5.69-13.44) and Thyroid stimulating hormone, TSH = 0.03 (Normal = 0.43-4.1) which was proven by a positive thyroid scan. His thyroid function normalized following the discontinuation of amiodarone. Investigations of his family history revealed that his father was known to have dilated cardiomyopathy (DCM) and was treated with AICD, but had a sudden cardiac death a few years ago while he was waiting for cardiac transplantation. In addition, two of his sisters had pacemakers inserted, and one of his paternal uncles died suddenly at the age of 25 years. Family pedigree is shown in (Figure 5).

## Discussion

**Figure 6:**
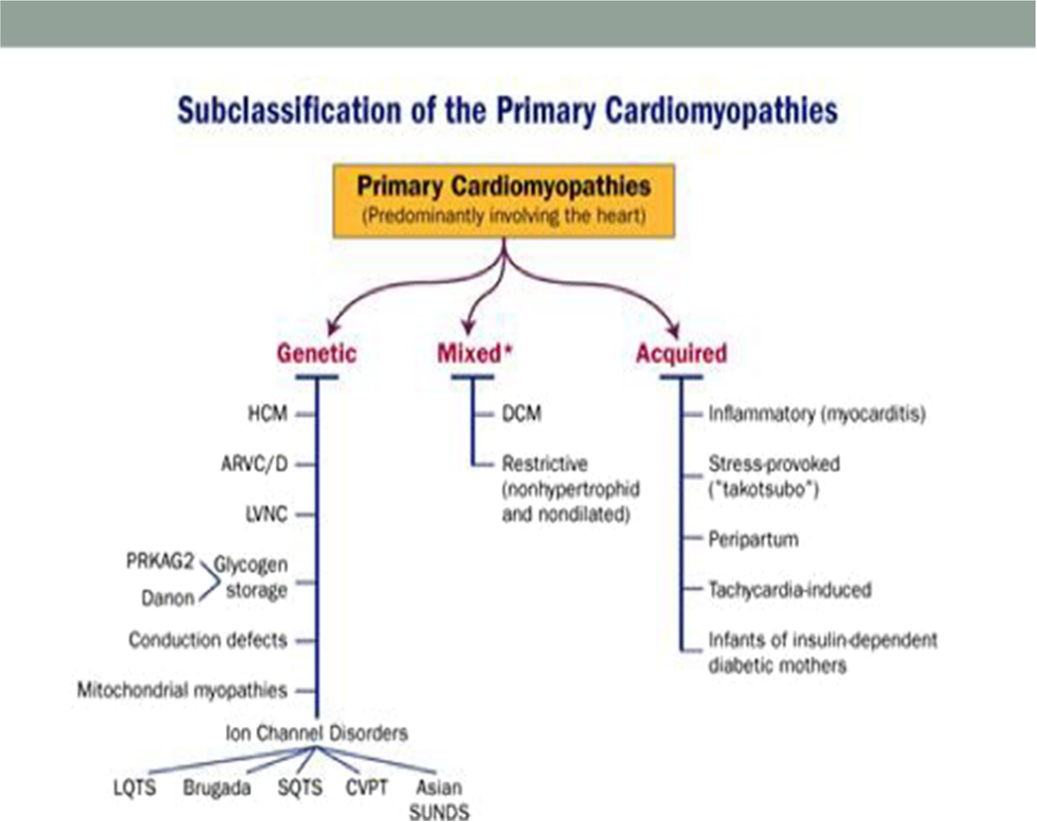
**General classification systems into primary cardiomyopathies according to the American heart association and the European society of cardiology.**

**Figure 7:**
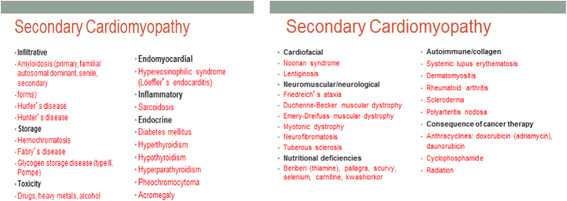
**General classification systems into secondary cardiomyopathies according to the American heart association and the European society of cardiology.**

Though the American heart association (AHA) and the European society of cardiology (ESC) defined and classified cardiomyopathies differently [7, 8]; however, both organizations have agreed on general classification systems into primary and secondary cardiomyopathies (Figures 6 and 7). While the AHA considers ion channelopathies as one of the cardiomyopathies, the ESC does not consider ion channelopathies as an accepted cardiomyopathy because genes encoding for ion channels might not result in morphofunctional phenotypes. Thus, in 2013, the American college of cardiology (ACC) and AHA defined cardiomyopathies as disorders characterized by morphologically and functionally abnormal myocardium in the absence of any other disease that is sufficient, by itself, to cause the observed phenotype [9]. The majority of these cases are secondary to coronary artery disease, hypertension and valvular cardiomyopathy. The number and severity of each of the traditional cardiovascular risk factors should be evaluated. These risk factors must evaluate as continuous rather than discrete variables that exert a dose-dependent effect on coronary artery disease. Moreover, there are novel biomarkers reflecting thrombosis, inflammation and oxidative stress that contribute to the pathophysiological process of atherthrombosis in acute ischemic heart disease [10]. A fourth of the cases of congestive cardiac failure are secondary to idiopathic cardiomyopathy [11]. It is possible that idiopathic dilated cardiomyopathy (IDC) represents a common expression of myocardial damage that has been produced by un-established myocardial insults [12]. The incidence of a familial form of dilated cardiomyopathy (is up to 20% of all cases of DCM [13, 14]. Identifying these individuals will help the treating physician in pursuing major changes in the clinical management or counseling of these patients with the following recommendations:

1. Avoidance of competitive sport activity.
2. Instituting early medical treatment such as ACEi and β blockers.
3. Refraining from the use of injurious agents:
  A. Succinyl choline or volatile anesthetics (halothane and isoflurane) in emerinopathies and laminopathies.
  B. Statins in genetic cardiomyopathies with possible involvement of the skeletal muscle, even when markers of myopathy are negative.
  C. Patients with mitochondrial cardiomyopathy and epilepsy should not receive valproate because it may cause pseudo atrophy of brain.

**Figure 8:**
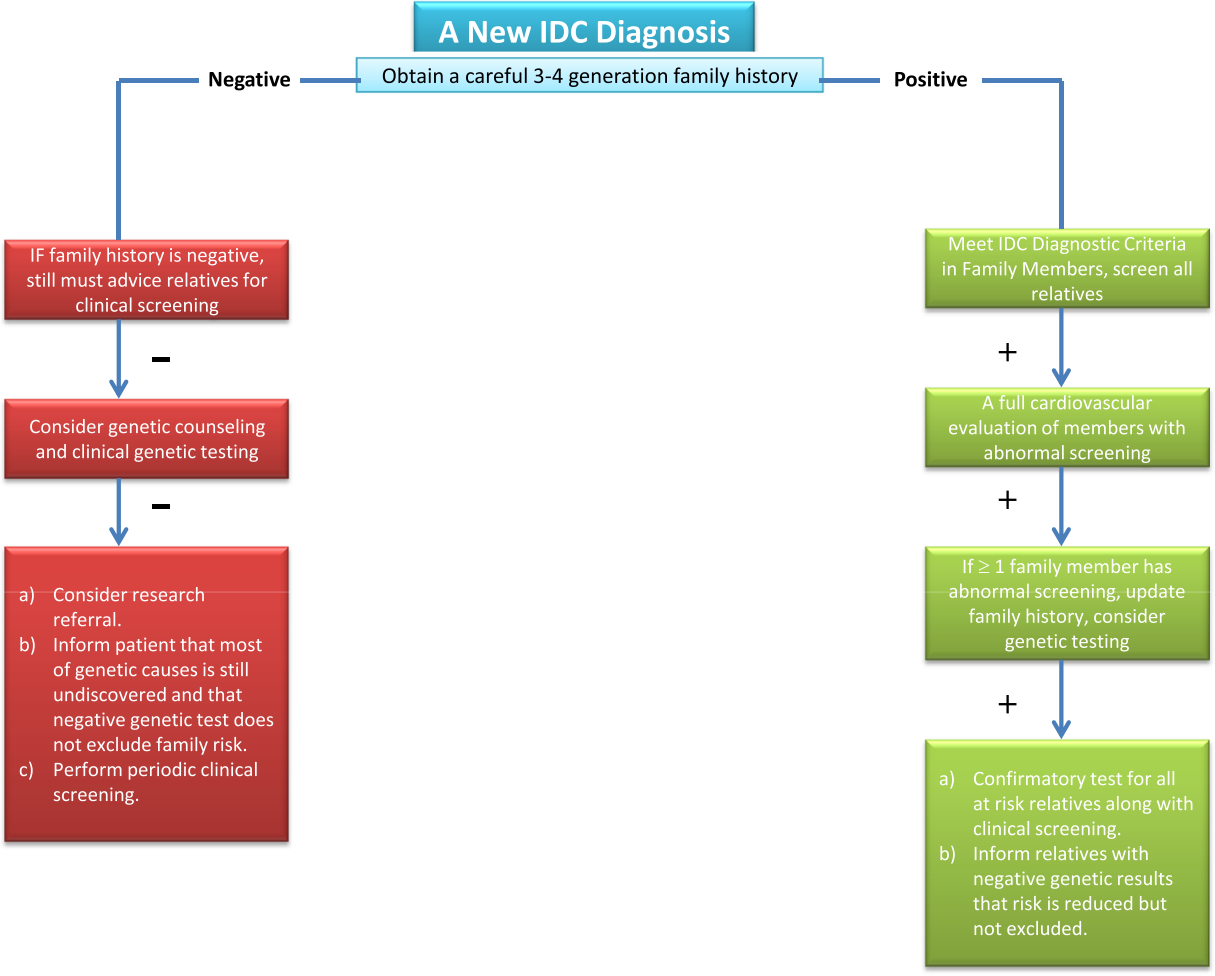
**Flow diagram of genetic risk assessment for patients newly diagnosed with idiopathic dilated cardiomyopathy or familial dilated cardiomyopathy.**

Here we differentiate a patient with ‘true’ IDC who had his first-degree family members clinically screened (history, physical examination, echocardiogram, and ECG) to rule out familial dilated cardiomyopathy (FDC) versus a ‘presumptive’ IDC – one who is negative for familial disease by a careful 3–4 generation family history but has not had family members screened beyond the family history [15]. Our patient fulfilled the criteria for idiopathic cardiomyopathy that had first-degree family members with a definite diagnosis of idiopathic dilated cardiomyopathy (IDC). Despite the evidence supporting a genetic basis of IDC/FDC, physicians have poorly complied with the implementation of guidelines [16]. Adherence to such guidelines would require a shift in focus from strictly therapeutic measures for a single patient presenting with advanced disease to the consideration and assessment of DCM risk for an entire family. This represents a major health problem in the Kuwaiti society and the middle-eastern communities in general. In this regard, close relative marriages are common in these societies, which results in the cluster of pathological genes in certain families. This is also seen in inherited hematological disorders. Usually families affected by these disorders end up having to live with the potential tragedy of losing their off springs or close relatives before their eyes. Familial cardiomyopathy is a serious disease when the phenotypic expression leads to severe cardiomyopathy, and our patient represents an example of this serious disease. The patient became handicapped and lost his father, who was his caregiver. Therefore, a better understanding the genetic basis of FDC disease in Kuwait will help medical staff convince families to change the pattern of familial marriage. This change will save lives and prevent families from going through such a devastating experience and will help them enjoy having healthy generations. We sought to adhere to the guidelines in pursuing the genetic work up of the family of our patient according to the flow diagram shown in (Figure 8). Although, numerous genes have been reported in association with non-syndrome dilated cardiomyopathy [9, 16], there have been no reports of genes in association with DCM in Kuwait.

## Current and future prospective

Genetic polymorphisms associated with cardiovascular responses were reported in number of studies. Three main genes were shown as key players in the regulation of cardiac responses causing electrophysiology and cardiac myocyte hypertrophy. These genes are calcium/calmodulin-dependent kinase IV (CaMKIV), the G-protein-coupled receptor kinases (GRKs) and the heptahelical G-protein-coupled receptors (GPCRs) [17–19]. The family history of the patient was taken and clinical screening of first-degree relatives (who are at risk) was performed. A total of 26 blood samples from all first-degree family members were collected, shown in the pedigree (Figure 5). The mode of inheritance in this family appears to act through an autosomal dominant type. Whole exome sequencing using Hiseq technology was our method of choice to unravel the causative genes, through a series of phenotype-genotype filtration processes. A thorough evaluation of the patients’ relatives who are at risk will be performed to detect the absence or presence of pathologic mutation(s).

## Study limitations

The patient is assumed to have had “idiopathic cardiomyopathy” based on the absence of secondary causes; however, this assumption may be inaccurate. The patient had an echocardiogram 6 months prior to hospital admission and the left ventricular function was reported to be normal. Inherited DCM is a slow progressive disease in which the left ventricular systolic function deteriorates over a number of years rather than over few months. Hence, this case lacks the documentation of the progressive nature of the disease. Moreover, he had an arrhythmic sudden cardiac death, which required prolonged cardiopulmonary resuscitation. Actually, it took the paramedics 5 minutes to arrive at the scene and therefore, this prolonged ischemic insult may have caused myocyte necrosis resulting in permanent left ventricular systolic dysfunction. Finally, rare causes of DCM cannot be ruled out, as myocardial biopsy was not available in Kuwait at that time.

## Conclusions

Familial cardiomyopathies are genetic diseases and are becoming widely recognized worldwide. The phenotypic expression is linked to the specific causative genotype in a small percentage of dilated cardiomyopathy of unknown etiology. To better understand the phenotypic expression, treating physicians need to study the genetic pedigree of affected patients with suspected inherited cardiomyopathy. This testing may affect the management of the patients carrying the defective gene as well as their first-degree relatives. However, the results of the genetic testing must be correlated with the clinical presentation, as it is too early to take clinical decisions based on the genetic study alone.

## Consent

Ethical approval from Dasman Diabetes Institute Ethical Review Committee and written informed consent was obtained from the patient’s next of kin as well as from all family members who participated in the molecular genetic of this study. Written approval from the patient’s next of kin was also obtained for publication of this Case Report and any accompanying images. A copy of the written consent is available for review by the Editor-in-Chief of this journal.

## Abbreviations

ACC: American college of cardiology
AHA: American heart association
AICD: Automated implantable cardioverter-defibrillator
CaMKIV: Calcium/calmodulin-dependent kinase IV
CTS: Computed tomography scan
DCM: Dilated cardiomyopathy
ECG: Electrocardiography
EEG: Electroencephalograph
ESC: European society of cardiology
FDC: Familial dilated cardiomyopathy
FT4: Thyroid test
GPCRs: G-protein-coupled receptors
GRKs: G-protein-coupled receptor kinases
ICU: Intensive care unit
IDC: Idiopathic dilated cardiomyopathy
LBBB: Left bundle branch block
LV: Left ventricular
LVEF: Left ventricular ejection fraction
MRI: Magnetic resonance imaging
TSH: Thyroid stimulating hormone
WHO: World Health Organization.
